# Spectrum of podocytopathies in new-onset nephrotic syndrome following COVID-19 disease: a report of 2 cases

**DOI:** 10.1186/s12882-020-01970-y

**Published:** 2020-08-04

**Authors:** Rajib K. Gupta, Ramya Bhargava, Al-Aman Shaukat, Emily Albert, John Leggat

**Affiliations:** 1 Department of Pathology, SUNY Upstate Medical University, 750 E Adams Street, Syracuse, NY 13210 USA; 2 Present: Department of Pathology, Louisiana State University Health Sciences Center, Shreveport, LA USA; 3 Department of Nephrology, SUNY Upstate Medical University, 750 E Adams Street, Syracuse, NY 13210 USA; 4 Department of Medicine, SUNY Upstate Medical University, Syracuse, NY USA

**Keywords:** COVID-19, Nephrotic syndrome, Podocytopathy, Collapsing glomerulopathy

## Abstract

**Background:**

Coronavirus disease-2019 (COVID-19) is an ongoing pandemic which has affected over 12 million people across the globe. Manifestations in different organs systems are being reported regularly. Renal biopsy findings in hospitalized COVID-19 patients presenting solely with acute kidney injury (AKI) have recently been described in published literature in few case reports. The findings include diffuse acute tubular injury (ATI) along with the glomerular lesion of collapsing glomerulopathy (CG). However, nephrotic syndrome as the presenting complaint of COVID-19 has not been reported widely, neither has any other glomerular lesion other than CG.

**Case presentation:**

We describe the kidney biopsy findings of two patients who had recent diagnoses of COVID-19 and presented with new-onset nephrotic syndrome. Renal biopsy in both patients showed ATI (as in previous reports) and distinct glomerular findings on light microscopy – that of minimal change disease (MCD) initially in one patient followed by CG in a subsequent biopsy and CG at the outset in the other patient. The electron microscopic findings in both patients were that of severe podocytopathy (diffuse and severe podocyte foot process effacement).

**Conclusion:**

Our cases highlight a novel clinical presentation of COVID-19 renal disease, not described before, that of new-onset nephrotic syndrome. While all published case reports describe CG as the glomerular pathology, we describe a non-CG pathology (MCD) in one of our cases, thereby adding to the repertoire of renal pathology described in association with COVID-19 patients. However, the exact mechanism by which podocyte injury or podocytopathy occurs in all such cases is still unknown. Optimal treatment options for these patients also remains unknown at this time.

## Background

COVID-19 currently affects over 13 million people across the world and has caused over 500,000 deaths since it was recognized in Wuhan, China [1]. While primarily a respiratory pathogen, acute kidney injury (AKI), has been reported in hospitalized patients, in addition to hematuria and proteinuria [2]. AKI in these patients is associated with increased severity of the illness. Renal histopathology has been studied in these patients mainly by post-mortem studies and more recently, via few antenatal renal biopsy-based case reports. While AKI has been described in the setting of multi-organ failure in CoViD-19, nephrotic syndrome as the presenting complaint of COVID-19 has not been described. We herein describe two cases where the patients presented primarily with nephrotic syndrome with a temporal association with COVID-19; the two renal biopsies showed two different histologic lesions on light microscopy (at least on initial biopsy) with diffuse podocytopathy as the sole ultrastructural lesion for both cases. We then surmise the possible mechanisms of these injuries and explore options for therapy.

## Case presentation

### Case 1

**Fig. 1:**
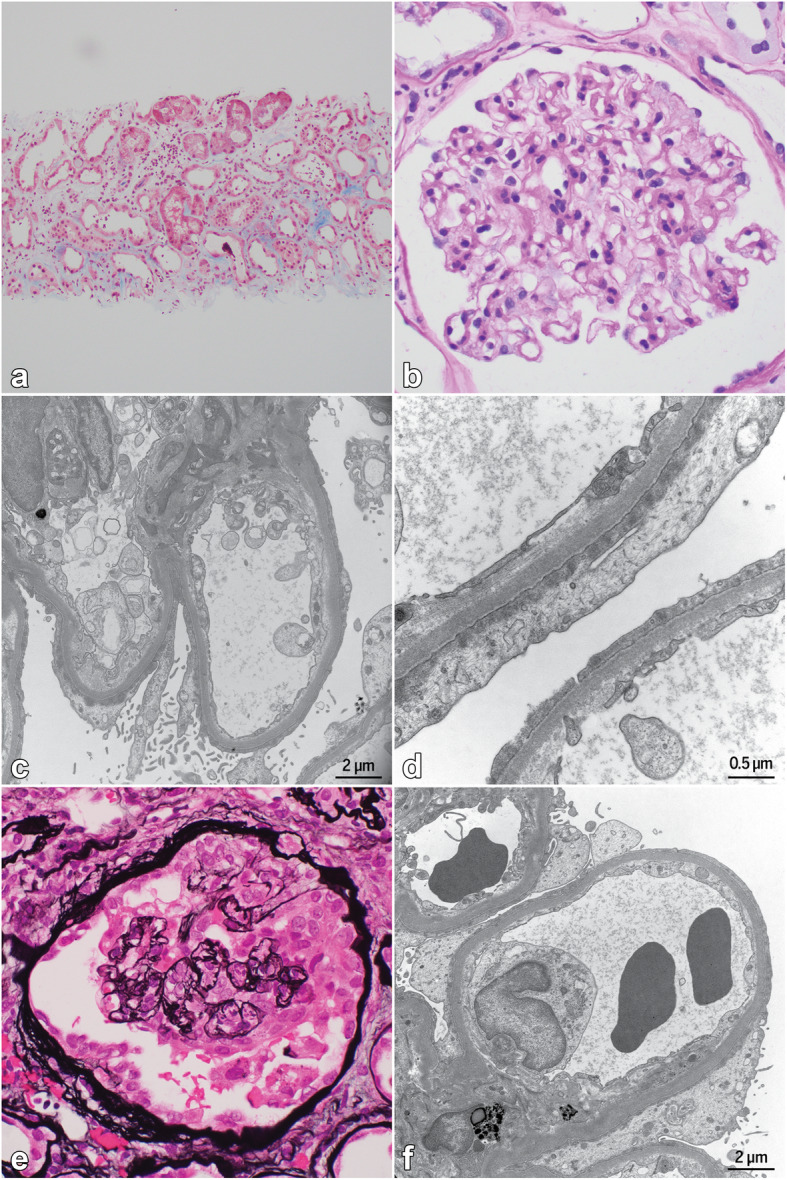
Images of case 1. **a-d**. First biopsy of case 1 – **a**. Biopsy core showing diffuse acute tubular injury in the form of epithelial flattening, intra-luminal dilatation and variably prominent tubular cell nuclei; background interstitium shows fibrosis intermixed with edema (Masson trichrome stain; original magnification, × 100); **b**. Representative glomerulus from the biopsy appearing completely unremarkable (PAS stain; original magnification, × 400); **c**. electron microscopy view of one glomerulus showing complete foot process effacement over adjacent capillary loops (original magnification, × 6800); and **d**. Electron microscopy view showing complete foot process effacement over 2 facing capillary loops (original magnification, × 18,500); **e-f**. Second biopsy of case 1 - **e**. Representative glomerulus showing global collapse of capillary loops associated with circumferential podocyte capping and podocyte hypertrophy/hyperplasia (Jones methenamine silver; original magnification, × 400), and **f**. Electron microscopy view of one glomerulus showing complete foot process effacement over adjacent capillary loops (original magnification, × 6800)

A 71-year-old Asian Indian male presented on 04/29/2020 with a two-week history of progressive swelling of both lower limbs, excessive daytime sleepiness, lethargy, lack of taste sensation and metallic taste in his mouth. He had reduced urine output but no hematuria, fever, sore throat or cough. His past medical history included type 2 diabetes on oral hypoglycemics, hypertension and benign prostatic hypertrophy. His medications included benazapril, aspirin, amlodipine and carvedilol. He was not a smoker and did not have any family history of renal disease. On review of his medical records, his baseline creatinine was 1.19 mg/dL and urine microalbumin-creatinine ratio (MAC) was 197 in October 2018. On examination, his blood pressure (BP) was 150/92 mmHg, heart rate (HR) 82/min and regular, body temperature 36.9 °C, oxygen saturation 97% on room air, and 3+ pedal edema. Heart sounds, breath sounds and abdominal palpation were normal. Blood tests revealed hemoglobin (Hb) 12.9 g/dL, white blood cell (WBC) count 7.2*10^3^/μL, platelets 207*10^3^/μL, sodium 124 mmol/L, potassium 5.5 mmol/L, bicarbonate 15 mmol/L, blood urea nitrogen (BUN) 33 mg/dL, creatinine 4.49 mg/dL and albumin 2.0 g/L. Urinalysis (UA) showed no RBCs or casts but heavy proteinuria > 500. Urine protein creatinine ratio (UPCR) was 18.46 g/g; a 24-h urine collection confirmed 16 g of protein in 1800 cc of urine. Serology revealed normal complements and negative levels for ANA, ANCA and anti-PLA2R antibody. Serologies for HIV, hepatitis C and hepatitis B were also negative. Serum and urine protein electrophoresis (SPEP and UPEP) did not show any paraproteins. IgG antibodies to SARS-CoV-2 were detected by Abbott Architect assay. Real-time SARS-CoV-2 PCR (RT-PCR) was performed using Cepheid Xpert Xpress assay and was positive. The diagnosis was new-onset nephrotic syndrome with AKI and he was commenced on intravenous furosemide. Based on the serology, further questioning revealed in mid-March he had developed a severe headache and myalgias for which he had taken ibuprofen for 3 days. He underwent a renal biopsy on 5th May. The biopsy showed 9 glomeruli, of which 3 were globally sclerosed. The patent glomeruli appeared completely unremarkable on light microscopy and did not show any hypercellularity, capillary loop thickening, collapse, crescents or podocyte hypertrophy. The tubulo-interstitium showed moderate scarring intermixed with edema, along with diffuse acute tubular injury/necrosis (ATI/ATN). Interstitial inflammation was patchy, mild and predominantly lymphoplasmacytic. Direct immunofluorescence study was negative. Electron microscopy (EM) of 2 glomeruli revealed diffuse podocyte foot process effacement (Fig. 1a-d). Final diagnosis was consistent with minimal change disease (MCD) and ATI/ATN, likely secondary to recent CoVID-19 infection. The patient was continued on optimum doses of furosemide, benazapril and spironolactone. BP was controlled to 128/84 mmHg. Follow-up: Two subsequent COVID-19 RT-PCR tests came back negative on the 4th and the 5th of May. He was commenced on oral prednisone 60 mg a day on the 8th of May. Unfortunately, there was no response to high-dose steroids and the patient continued to have nephrotic-range proteinuria and worsening renal function. He was commenced on dialysis on 6/12/2020 for symptomatic uremia and fluid overload. He had a repeat renal biopsy was performed on June 30. The biopsy showed up to 7 glomeruli, of which 2 glomeruli were globally sclerosed. Of the remaining patent glomeruli, 1–2 glomeruli showed global collapse of capillary loops associated with podocyte capping and variable degree of podocyte hypertrophy/hyperplasia, consistent with collapsing change (collapsing-type FSGS), while most of the remaining glomeruli showed variable chronic ischemic changes in the form of capillary tuft collapse and Bowman’s capsule fibrosis; no glomerular crescents or other active (proliferative) lesions were seen in any of the glomeruli. The tubulointerstitium showed diffuse ATN changes similar to the first biopsy in the background of moderate to severe interstitial scarring. Few arteries sampled showed severe fibrointimal thickening. Immunofluorescence study was negative as before and EM showed endothelial cell swelling in some of the loops associated with diffuse podocyte foot process effacement (PFPE) as before (Fig. 1e-f). The final diagnosis was rendered as collapsing glomerulopathy (CG), ATN and arteriosclerosis.

### Case 2

**Fig. 2:**
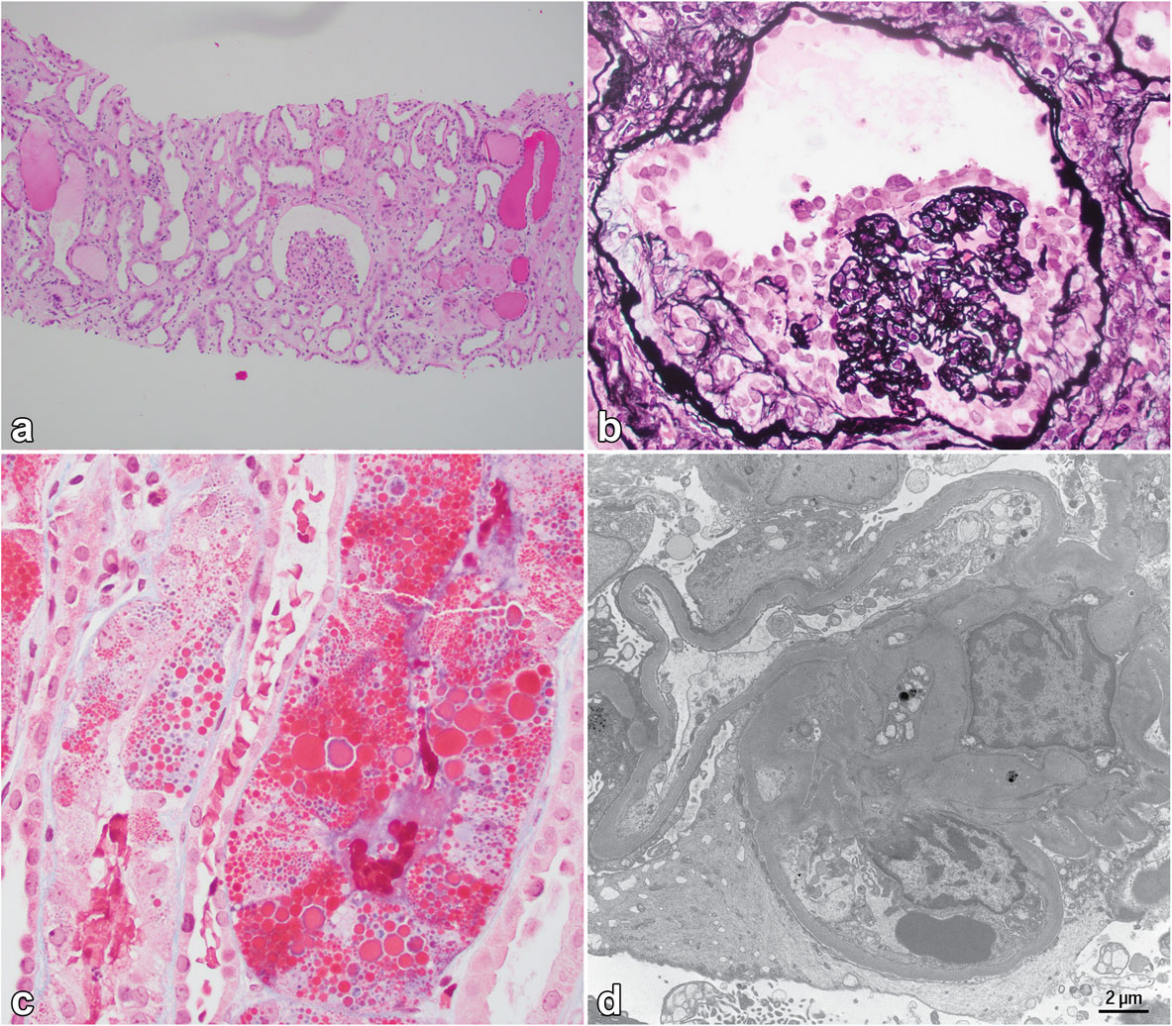
Images of case 2. **a**. Biopsy core showing diffuse acute tubular injury, few tubules with microcystic change and a single glomerulus with slight capillary collapse and podocyte capping (H&E stain; original magnification, × 100); **b**. A representative glomerulus from the biopsy showing global collapse of capillary loops associated with podocyte hypertrophy/hyperplasia (Jones methenamine silver; original magnification, × 400); **c**. A profile of proximal tubule in one of the biopsy cores showing abundant bright protein reabsorption droplets within the tubular epithelial cytoplasms (Masson trichrome stain; original magnification, × 400); and **d**. Electron microscopy view showing complete foot process effacement over adjacent capillary loops (original magnification, × 4800)

A 54-year-old African-American (AA) male presented on 04/30/2020 with a 2-week history of progressive swelling of both lower limbs, abdominal pain and nausea. Urine output was reduced but he had no hematuria. He had recently been admitted to the hospital from 04/06/2020 to 04/09/2020 with COVID-19 associated with fever, shortness of breath and AKI with a creatinine of 4.69 mg/dL; the AKI resolved with intravenous fluids, and his creatinine at the time of initial discharge was 1.29 mg/dL. His past medical history included type 2 diabetes on oral hypoglycemics and hypertension for which he was not on medication. His medications included metformin and calcium supplements. He was a smoker and did not have family history of renal disease. On review of his records, his baseline creatinine was 1.08 mg/dL in September 2019 and minimal proteinuria on urinalysis at 30 on the 04/07/2020. On examination on 4/30/20, his BP was 166/108 mmHg, HR 82/min and regular, body temperature 36.9 °C, oxygen saturation 96% on room air and 2+ edema in both his lower limbs up to his abdomen. His heart sounds and breath sounds were normal. Abdominal palpation revealed only mild tenderness around the umbilicus with no guarding or rigidity. Blood tests revealed Hb 12.1 g/dL, WBC count 5.0*10^3^/μL, platelets 203*10^3^/μL, sodium 142 mmol/L, potassium 4.4 mmol/L, bicarbonate 23 mmol/L, BUN 36 g/L, creatinine 4.67 g/L, and albumin 1.6 g/L. UA showed no RBCs casts but proteinuria and a UPCR of 16 g/g. He was found to be negative for ANA, ANCA, rheumatoid factor and anti-PLA2R antibody. HIV, hepatitis C and hepatitis B serology were all negative. COVID-19 RT-PCR was performed using Cepheid Xpert Xpress SARS-CoV-2 assay and was positive on the 1st of May and again on the 5th of May. The patient underwent a renal biopsy on the 5th of May. The biopsy showed up to 18 glomeruli of which none were globally sclerosed. Twelve out of 18 glomeruli showed changes consistent with collapsing-type FSGS. The interstitium appeared similar to case 1 in showing a diffuse ATN change amidst moderate scarring and slight edema. Few tubules also showed microcystic change, while some other tubules showed prominent intracytoplasmic reabsorption protein droplets. Interstitial inflammation was again mild and patchy. Immunofluorescence study was again negative, except for some segmental C3 trapping in some of the glomeruli. EM of a single glomerulus showed diffuse PFPE as in case 1 (Fig. 2), along with reduced capillary lumina, mild endothelial cell swelling (in few loops) and hypertrophic podocytes in the Bowman’s space. The final diagnosis was given as CG and ATN, secondary to COVID-19 infection. Follow-up: The patient was treated with diuresis with bumetanide and spironolactone, and his edema resolved. He was commenced on apixaban for thromboprophylaxis. As of 27th May, he continues to be RT-PCR positive. Attempts to recall the patient for APOL1 testing were futile and the patient is currently lost to follow-up.

In both cases, intracellular spherical structures resembling coronavirus were seen within the podocyte cytoplasms on EM, but they were later confirmed to be clathrin-coated vesicles (via personal communication with the pathology division of Infectious Diseases Pathology Branch, CDC, Atlanta, USA by one of the authors RKG).

## Discussion and conclusion

We describe 2 cases of new-onset podocytopathy causing nephrotic syndrome in type 2 diabetics with mild diabetic kidney disease where the onset had a temporal correlation with COVID-19. The first patient did not have any acute symptoms of COVID-19 infection but presented with nephrotic-range proteinuria and symptoms of uremia. The second patient had symptomatic COVID-19 infection and presented 4 weeks later with new-onset nephrotic syndrome. Both had ATI/ATN and variable degree of podocytopathy (MCD followed by CG in the first patient and CG at the outset in the second patient, along with diffuse PFPE on electron microscopy in both).

COVID-19 currently has affected about 13 million people across the world and has caused over 500,000 deaths since it was recognized in Wuhan, China [1]. While primarily a respiratory infection, the SARS-CoV-2 has been reported to result in proteinuria, hematuria and AKI from early reports from Wuhan, China [2]. A recent observational study of 5700 patients from New York area hospitals reported the need for renal replacement in 3.2% of the hospitalized COVID-19 patients [3]. The first histological analysis of post-mortem renal biopsies of 26 patients from China showed acute tubular injury, collapsing glomerulopathy, vascular injury and podocyte foot process effacement [4]. Four subsequent case reports (Table 1) in which patient presented with AKI, all reported ATI/ATN and CG [5–8]. Our case report describes a rare presenting complaint of COVID-19 patients with kidney involvement – that of new-onset nephrotic syndrome. Interestingly, the Asian-Indian patient (case 1) presented initially with minimal change-type glomerulopathy which progressed to CG with time, hinting at the possibility that non-AA and/or asymptomatic COVID-19 patients may pass through a more benign glomerular phase (as pointed out by Couturier et al. [8]) before developing florid CG, while genetic predisposition [6] like APOL-1 positivity coupled with symptomatic COVID-19 may push patients of African-American ethnicity to develop more severe glomerular pathology (that of CG) from the outset (Table 1).

**Table 1:** Chart enumerating details of 4 recent case reports of COVID-19 patients who had renal biopsies. M male, AA African-American, S. Cr serum creatinine, UPCr urine protein-creatinine ratio, DM diabetes mellitus, HTN hypertension, AKI acute kidney injury, CKD chronic kidney disease, UA urinalysis, ATI acute tubular injury, ATN acute tubular necrosis, LM light microscopy, IF immunofluorescence, Neg negative, CG collapsing glomerulopathy or collapsing-type focal segmental glomerulosclerosis, EM electron microscopy, ITEDD immune-type electron-dense deposits, APOL1 apolipoprotein 1

Published reports	Age/Sex/Ethnicity	Renal-associated signs & symptoms	Pre-existing disease(s)	Recent NSAID use	Renal biopsy findings	APOL1 gene testing
Larsen et al. [5] (1 case)	44/M/AA	AKI with S. Cr of 4.0 mg/dl, UA positive for blood and protein, spot UPCr 3.9 g/g	Poorly-controlled type 2 DM, HTN, dyslipidemia, CKD	Not known	LM: CG, ATI/ATN IF: Neg EM: severe FPE, no ITEDD present, occasional TRIs within glomerular endothelial cytoplasm	Positive
Peleg et al. [6] (1 case)	46/M/AA	AKI with S. Cr of 12.5 mg/dl, nephrotic-range proteinuria, hypoalbuminemia	Obesity, OSA	Yes (ibuprofen)	LM: CG, ATI/ATN, IF: Neg EM: Sample contained no glomeruli; tubules showed epithelial injury and protein reabsorption droplets but no virions	Positive
Kissling et al. [7] (1 case)	63/M/AA	AKI with S. Cr of 4.4 mg/dl, massive proteinuria (5 g/l) and hypoalbuminemia	HTN	Not known	LM: CG, ATI/ATN, IF: Neg EM: no ITEDD present, numerous spherical particles^a^ within podocyte cytoplasm and within intracytoplasmic vacuoles seen	Not done
Couturier et al. [8] (2 cases)	a. 53/M/AA b. 53/M/AA	a. AKI with S. Cr of 166 μmol/L (1.8 mg/dl), proteinuria and UPCr 564 mg/mmol (4.9 g/g) b. AKI with S. Cr of 470 μmol/L (5.3 mg/dl), proteinuria and 154.7 mg/mmol (1.3 g/g)	a. HTN b. HTN, untreated chronic hepatitis B	a. Not known b. Not known	a. LM: CG IF: Segmental glomerular deposits of IgM and C3 only EM: Not described b. LM: CG, ATI/ATN, IF: Segmental glomerular staining for C3 only EM: Not described	a. Positive b. Positive

^a^While the authors have described these as intra-cellular virions, few other authors have disputed these findings and have labeled them as normal intra-cellular organelles (see both Discussion and references [9] and [10])

The exact mechanism of podocyte injury in COVID-19 infection is still unknown, although several hypotheses have been put forward. The post-mortem study from China [4] has described SARS-CoV-2 like particles in the podocytes which showed podocyte effacement. Of the three case reports published [5–7], one found tubuloreticular inclusions (an indirect marker of viral replication and interferon overproduction) within endothelial cells [6], and spherical particles resembling virions, either free in podocyte cytoplasm or within podocyte cytoplasm vacuoles and suggest direct cytopathic effect of the virus, similar to HIV-associated glomerulopathy. This suggests that there may be a direct cytopathic effect resulting from the viral particles, similar to that of HIV-associated glomerulopathy. However, other experts have disputed these ultrastructural findings, and have pointed out that what these reports have described are more likely to be normal subcellular structures like clathrin-coated vesicles or multivesicular bodies [9, 10]. SARS-CoV-2 belongs to the beta-coronavirus family and utilizes the angiotensin-converting-enzyme 2 (ACE2) receptor to enter the target cells. These receptors are found in the proximal tubular epithelial cells and to a lesser extent in the podocytes which could explain the propensity of the virus to infect these cells [11]. Hua Su et al. [4] in their postmortem study, evaluated ACE2 immunohistochemical staining in the kidney samples of 5 patients, and found altered ACE2 staining in three patients, including prominent staining of the same in proximal tubular cells showing ATI, focal strong staining in parietal epithelial cells and occasional weaker staining in podocytes, compared to weak ACE2 staining in proximal tubular cells and no staining in the glomeruli in control archival kidney samples. Studies have shown that there is a specific renal tropism of the SARS-CoV-2 virus with preferential targeting of glomerular cells which might explain the widespread kidney injury even in patients without critical COVID-19 disease, as the patient in case 1 [12]. The mechanism causing the podocyte foot process effacement is likely to be mediated by multiple immunological pathways. Studies in non-COVID-19 related MCD have shown an imbalance in T-cell populations during the active phase of the disease with a prevalence of type 2 T-helper (Th2) cell cytokine profile (Th2; IL4,5,9,10 and 13) [13]. There is clinical evidence of response of MCD to B-cell depletion with rituximab, an anti-CD20 monoclonal antibody [14]. Interestingly, an important mechanism of tissue damage by COVID-19 infection is the generation of a cytokine storm. COVID-19 patients have been shown to have elevated levels of Th2-secreted cytokines such as IL4 and IL10 [15], which potentiate ARDS in the lungs. The serum levels of IL-2R and IL-6 in these patients are positively correlated with the severity of the disease [16]. The cytokine storm generating an immunological milieu with excessive production of Th2-generated cytokines as a trigger for the podocytopathy therefore remains a distinct possibility. This theory is further strengthened by other immunological manifestations such as cardiomyopathy and myocarditis in these COVID patients [17, 18]. While other immune mechanisms can result in immune complex deposition in the glomeruli leading to glomerulonephritides [19], neither of our patients nor the case reports of COVID-19 renal biopsies (Table 1) showed any immune complex deposition on immunofluorescence or on electron microscopy.

No specific treatment has been investigated for the treatment of COVID-19-associated MCD or CG. In non-COVID-19 MCD, high dose steroids are the mainstay of treatment [20], especially in childhood and steroid-sensitive MCD is known to respond to rituximab, an anti-CD20 monoclonal antibody [14]. It is not known if COVID-19 MCD responds to high-dose steroids, or when the steroid treatment needs to be initiated. In our first patient with MCD, we initiated high dose steroid therapy after he was found to be COVID-19 RT-PCR negative on 2 consecutive days. Vardhana et al. [21] categorically caution against starting immunosuppression in COVID-19 patients without a means of achieving viral control since an intact immune system is necessary to ensure viral clearance. It is not known if the patient will respond to high dose steroids. CG, on the other hand, is characterized by the absence of mature podocyte markers and patients with CG usually progress rapidly to end stage renal disease [22]. HIV-associated glomerulopathy is the other example of direct viral damage to the podocytes causing CG and is known to reverse and can be prevented by the use of retro-viral therapy [23]. When an effective therapy for SARS-CoV-2 becomes available, the effect may be similar on the COVID-19 related CG.

In conclusion, we present 2 cases of new-onset nephrotic syndrome in association with COVID-19 infection, one with MCD followed by CG in his second biopsy (non-AA patient) and another with CG in the first biopsy (AA patient). It is likely that just as in non-COVID-19-associated native biopsies [24], MCD and CG belong to a continuous spectrum of COVID-19 related podocytopathy caused by direct or cytokine-mediated toxicity from the SARS-CoV-2 virus. The exact mechanism of podocyte injury in COVID-19 diseases however still remains unknown and no treatment options have been shown to be completely effective.

## Data Availability

All the data supporting our findings is contained within the manuscript.

## Abbreviations

COVID-19: Coronavirus disease-2019
SAR-CoV-2: Severe respiratory syndrome coronavirus 2
APOL-1: Apolipoprotein L-1
MCD: Minimal change disease
CG: Collapsing glomerulopathy
